# “*In vitro*” Effect of Different Follicle—Stimulating Hormone Preparations on Sertoli Cells: Toward a Personalized Treatment for Male Infertility

**DOI:** 10.3389/fendo.2020.00401

**Published:** 2020-06-18

**Authors:** Iva Arato, Giuseppe Grande, Ferran Barrachina, Catia Bellucci, Cinzia Lilli, Meritxell Jodar, Maria Chiara Aglietti, Francesca Mancini, Federica Vincenzoni, Alfredo Pontecorvi, Riccardo Calafiore, Rafael Oliva, Giovanni Luca, Francesca Mancuso, Domenico Milardi

**Affiliations:** ^1^Department of Experimental Medicine, University of Perugia, Perugia, Italy; ^2^Research Unit on Human Reproduction, International Scientific Institute Paul VI, Rome, Italy; ^3^Division of Endocrinology, Fondazione Policlinico Universitario “Agostino Gemelli”, Rome, Italy; ^4^Molecular Biology of Reproduction and Development Research Group, Institut d'Investigacions Biomèdiques August Pi i Sunyer (IDIBAPS), Department of Biomedical Sciences, Faculty of Medicine and Health Sciences, University of Barcelona, Barcelona, Spain; ^5^Biochemistry and Molecular Genetics Service, Hospital Clínic, Barcelona, Spain; ^6^Department of Medicine, University of Perugia, Perugia, Italy; ^7^Biochemistry and Clinical Biochemistry Institute, School of Medicine, Catholic University of Rome, Rome, Italy; ^8^Department of Laboratory Diagnostic and Infectious Diseases, Fondazione Policlinico “A. Gemelli” IRCCS, Rome, Italy; ^9^Division of Medical Andrology and Endocrinology of Reproduction, University of Perugia and Saint Mary Hospital, Terni, Italy

**Keywords:** Sertoli cells, alpha follitropin, beta follitropin, urofollitropin, proteomic analysis

## Abstract

Follicle-stimulating hormone (FSH), a major regulator of spermatogenesis, has a crucial function in the development and function of the testis and it is extensively given as a fertility treatment to stimulate spermatogenesis. We analyzed the effects of different FSH preparations (α-follitropin, β-follitropin, and urofollitropin) in combination with testosterone on porcine pre-pubertal Sertoli cells. To study the effect of the different FSH treatments in the Sertoli cell function we performed Real Time PCR analysis of AMH, inhibin B, and FSH-r, an ELISA assay for AMH and inhibin B, and a high-throughput comparative proteomic analysis. We verified that all three preparations induced a reduction of AMH in terms of mRNA and secreted proteins, and an increase of inhibin B in terms of mRNA in all the FSH formulations, while solely α-follitropin produced an increase of secreted inhibin B in the culture medium. Comparative proteomic analysis of the three FSH preparations identified 46 proteins, 11 up-regulated and 2 down-regulated. Surprisingly, the combination of testosterone with β-follitropin specifically induced an up-regulation of eight specific secreted proteins. Our study, showing that the three different FSH preparations induce different effects, could offer the opportunity to shed light inside new applications to a personalized reproductive medicine.

## Introduction

Follicle-stimulating hormone (FSH), a glycoprotein hormone secreted by the anterior pituitary gland, plays a key function in the treatment of human infertility. In infertile women it is widely prescribed to stimulate follicular development, meanwhile, in males, it is used alone or in association with Human chorionic gonadotropin (hCG) to trigger off and maintain spermatogenesis both in hypogonadotropic hypogonadism (1), and in oligozoospermic subjects with normogonadotropic hypogonadism (2).

FSH comprises two subunits, α and β, which are both glycosylated and contain four N-linked carbohydrates. The different content in sialic acid at the C-terminal determines a family of glycoforms that explain the structural and functional heterogeneity of the different FSH formulations (3).

The preparations of FSH available in the market are derived by either recombinant DNA technology (rFSH such as α- and β-follitropin) or post-menopausal urines (urofollitropin). α- and β-follitropins are synthesized by the same recombinant technology, producing identical dimeric α-FSH and β-FSH subunits, but with differences in the further glycosylation and in the procedures of purification. In contrast, urofollitropin consists of FSH with a minimal LH activity, and it has low specific activity (~100–150 IU FSH/mg protein). The low specific activity of this preparation could be explained by the fact that more than 95% of the protein content correspond to non-specific co-purified urinary proteins (1).

Regarding the efficacy of the different FSH preparations in the female, many contradictory results have been published in the last 2 decades. The meta-analyses regarding the clinical efficacy of different FSH preparations demonstrated no significant differences in clinical or ongoing pregnancy and in the live-birth rate, in the miscarriage rate, or for the incidence of multiple pregnancy rate or ovarian hyperstimulation syndrome (OHSS) between rFSH and urofollitropin (4–8).

Up to now, in the male, no data exist regarding the efficacy of the treatment in relation to the FSH-therapy used. However, a meta-analysis reported a significant positive effect of the treatment with FSH both on sperm parameters and on pregnancy rate in oligozoospermic patients with normal FSH levels (9). Unfortunately, the studies included in the meta-analyses have an extremely heterogeneity in the selection criteria of the patients, in primary and secondary end-points, in the doses of FSH treatment and in time of treatment (10).

Sertoli cell (SC) is one of the principal actors in spermatogenesis as it provides nourishment, and structural and functional support to germ cells. Moreover, it protects germ cells by the blood-testis barrier (BTB) and by the production of immunomodulatory factors (11). In testis, FSH controls the function of SC through FSH receptors (FSH-r), which are only present in SC. In particular, FSH plays a pivotal role in the early stages of spermatogenesis, while testosterone has a major role in spermiogenesis (12).

In the prepubertal testis, SC is the most representative cell population. However, during this stage, there is a low activity of the hypothalamic–pituitary–gonadal axis reflected by the high levels of Anti-Müllerian Hormone (AMH) and inhibin B in serum (13). In contrast, during puberty, testosterone induces SC maturation and inhibits AMH production (13).

The aim of this research was to assess the effects of the different FSH formulation in an “*in vitro*” model of porcine pre-pubertal SC, in order to evaluate the SC responsiveness to pharmacological treatment of different FSH preparations, never assessed until now.

## Materials and Methods

### SC Culture, Characterization, and Stimulation

Pure porcine pre-pubertal SC were isolated and characterized according to previously reported methods (14–16).

Purified SC cultures, as previously stated (14–16), were treated for 48 h as follows, and stimulation was performed according to the previously described protocol (17):

- Stimulated with testosterone (0.2 μg/ml; SIT, Pavia, Italy) (control group);
- Stimulated with α-follitropin (α-FSH) 100 nM and testosterone (0.2 μg/ml);
- Stimulated with β-follitropin (β-FSH) 100 nM and testosterone (0.2 μg/ml);
- Stimulated with Urofollitropin (u-FSH) 100 nM and testosterone (0.2 μg/ml).

We used testosterone in addition to any of the FSH formulation to mimic a physiological condition in testis, considering that both FSH and testosterone are essential for the adequate spermatogenesis (12).

### Quantitative, Real-Time PCR

Analyses for AMH, inhibin B, and FSH receptor (FSH-r) were performed as previously described (17) employing the primers listed in Table 1. Total RNA was extracted from SC monolayers obtained in the experimental groups using Trizol reagent (Sigma-Aldrich, Milan, Italy), and quantified by reading the optical density at 260 nm. In detail, 2.5 μg of total RNA was subjected to reverse transcription (RT, Thermo Scientific, Waltham, MA, USA) to a final volume of 20 μl. We performed the qPCR with the use of 25 ng of the cDNA obtained by RT and a SYBR Green Master Mix (Stratagene, Amsterdam, The Netherlands). This procedure was performed in an Mx3000P cycler (Stratagene), using FAM for detection and ROX as the reference dye. We normalized the mRNA level of each sample against β-actin mRNA and expressed it as fold changes vs. the levels in the control group.

**Table 1:** Primer sequences for PCR analyses.

**Gene**	**Forward sequences (5^**′**^-3^**′**^)**	**Reverse sequences (5^**′**^-3^**′**^)**
AMH	GCGAACTTAGCGTGGACCTG	CTTGGCAGTTGTTGGCTTGATATG
Inhibin B	CCGTGTGGAAGGATGAGG	TGGCTGGAGTGACTGGAT
FSH-r	TGAGTATAGCAGCCACAGATGACC	TTTCACAGTCGCCCTCTTTCCC
β-actin	ATGGTGGGTATGGGTCAGAA	CTTCTCCATGTCGTCCCAGT

### Culture Media Isolation

Aliquots of the culture media (CM) of SC were collected after 48 h of stimulation, centrifuged at 1,500 g for 10 min, and the supernatant was saved at −20°C for proteomic analysis and for an ELISA assay for Inhibin B and AMH secretion performed as previously described (18).

### Proteomic Analysis of the SC Secretome

The SC CM for each sample was thawed and centrifuged at 3,000 g for 20 min at 4°C. The resulting supernatants were filtered (0.45 μm pore size) to remove cell debris and other impurities if any. Afterward, to perform protein solubilization, sodium dodecyl sulfate (SDS) and phenylmethylsulfonyl fluoride (PMSF) were added to a final concentration of 2% (w/v) and 1 mM, respectively. After 30 min, all samples were centrifuged at 4°C at 16,000 g for 10 min, and the supernatants (soluble proteins) were kept, and proteins were precipitated overnight in 80% cold acetone (v/v) at −20°C. After centrifugation at 17,530 g for 15 min at 4°C, the protein precipitates were collected and resuspended in 1% SDS, 1 mM PMSF in phosphate-buffered saline (PBS) to perform protein quantification using the BCA protein Assay Kit (Pierce™ BCA protein Assay Kit, Thermo Fisher Scientific, Rockford, IL), according to manufacturer's recommendations.

The TMT labeling was performed as described previously (19, 20) and according to the manufacturer's instructions. Briefly, 50 μg of proteins from each sample were transferred to a new tube and adjusted to a final volume of 50 μl with 100 mM triethylammonium bicarbonate (TEAB) to obtain a 1 μg/μl concentration. Proteins were reduced in 9.5 mM tris (2-carboxyethyl) phosphine (TCEP), alkylated with 17 mM iodoacetamide (IAA), and precipitated by adding six volumes of 100% cold acetone. Then, samples were centrifuged at 17,500 g, and the acetone-precipitated protein pellets (containing 50 μg of proteins) were resuspended in 50 μl of 100 mM TEAB. Trypsin was added at a 1:22 protease-to-protein ratio and incubated overnight at 37°C. Prior to peptide labeling, an aliquot from each sample was taken and combined at equal amounts to form the internal control. After, 35 μg of peptides from each sample (including the internal control) were labeled with TMT isobaric tags (TMT 10-plex Mass Tag Labeling; Thermo Fisher Scientific, Rockford, IL). The technical reproducibility and analytical reliability of the approach were assessed by performing duplicate analyses on the internal control. Then, 15 μl of the TMT label reagents, previously resuspended in acetonitrile anhydrous (ACN), were added to the corresponding sample, followed by 1 h incubation at RT. Afterward, the reaction was stopped by adding 5% hydroxylamine. The TMT-labeled samples were combined at equal amounts constituting one multiplex pool, which was dried in a vacuum centrifuge and resuspended in 50 μl of 0.5% trifluoroacetic acid in 5% ACN. After, labeled peptides were cleaned up via reversed-phase C18 spin columns (Pierce C18 Spin Columns, Thermo Fisher Scientific, Rockford, IL), according to manufacturer's instructions. Then, the peptides were reconstituted in 0.1% formic acid (FA) to be processed by LC-MS/MS.

Our MS data was collected using a nano-LC Ultra 2D Eksigent (AB Sciex, Brugg, Switzerland) attached to an LTQ-Orbitrap Velos (Thermo scientific, San Jose, CA). Peptides were injected onto a C18 trap column (L 2 cm, 100 μm ID, 5 μm, 120 Å; NanoSeparations, Nieuwkoop, the Netherlands) and chromatographic analyses were performed using an analytical column (L 15 cm, 75 μm ID, 3 μm, 100 Å; Thermo scientific, San Jose, CA). The buffers used for the analysis were buffer A (97% H_2_O-3% ACN, 0.1% FA) and buffer B (3% H_2_O-97% ACN, 0.1% FA). A peptide mixture was loaded onto the analytical column with the following gradient: time 0–5 min, 0% of B; 5–180 min, 0–32.5% of B; 180–185 min, 32.5–100% of B at a flow rate of 400 ml/min; and 185–200 min, 100% of B at a flow rate of 400 nl/min to avoid carry-over. MS/MS analyses were performed using an LTQ-Orbitrap Velos (Thermo Fisher Scientific, Waltham, MA) with a nanoelectrospray ion source. The LTQ-Orbitrap Velos settings included one 30,000 resolution at 400 m/z MS1 scan for precursor ions followed by MS2 scans of the 15 most intense precursor ions, at 30,000 resolution at 400 m/z, in positive ion mode. The lock mass option was enabled, and mass calibration was performed on polysiloxane (m/z 445.12003). MS/MS data acquisition was completed using Xcalibur 2.1 (Thermo Fisher Scientific, Waltham, MA). MS2 experiments were performed using higher-energy collision dissociation (HCD) with a normalized collision energy of 42%.

LC-MS/MS data was analyzed using Proteome Discoverer 1.4.1.14 (Thermo Fisher Scientific, Waltham, MA, USA) based on SEQUEST HT cluster as search engine (University of Washington, licensed to Thermo Electron Corp., San Jose, CA) against UniProtKB/Swiss-Prot database with Sus scrofa (released September 2018; 3,339 sequences). Searches were run applying the following parameters: two maximum missed cleavage sites for trypsin, TMT-labeled lysine (+229.163 Da) and methionine oxidation (+15.995 Da) as dynamic modifications, cysteine carbamidomethylation (+57.021 Da) as a static modification, 20 ppm precursor mass tolerance, 0.6 Da fragment mass tolerance. Percolator was used for protein identification, applying the following identification criteria: at least one unique peptide per protein and a FDR of 1%. The reporter ion intensities were corrected according to the isotopic purities provided by the manufacturer.

For protein quantification purposes, only unique peptides were used, and protein ratios (i.e., TMT-127/TMT-126) were normalized to protein median. The cut-off for up-regulated proteins was ≥1.500, and for down-regulated proteins was ≤ 0.667 as previously reported (21).

### Statistical Analysis

Values reported in the figures are the mean ± S.D. of three independent experiments, each one performed in triplicate. Statistical analysis was performed by the paired Student's *t*-test using SigmaStat 4.0 software (Systat Software Inc., CA, USA). All tests were performed in triplicate, and statistically significance was assigned for *p* < 0.05.

## Results

### Purification and Characterization of SC

**Figure 1:**
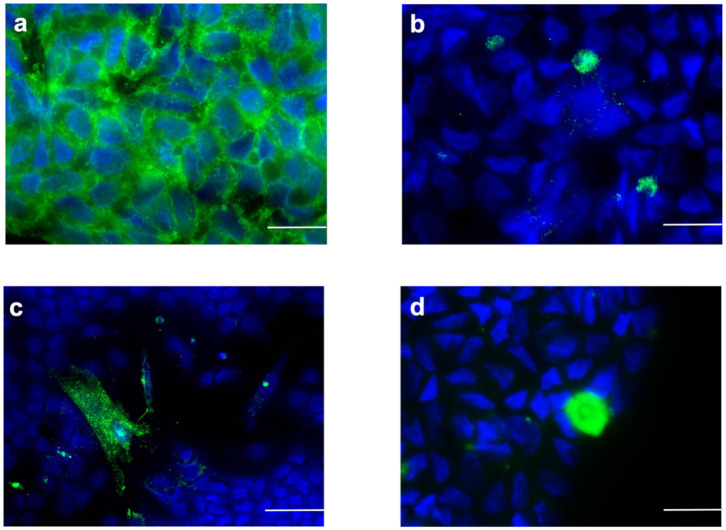
Sertoli cells characterization by immunofluorescence staining (green color). Sertoli cell monolayers were characterized by the expression of **(a)** AMH, **(b)** INSL3, **(c)** ASMA, and **(d)** (PGP9.5). Nuclei are labeled with DAPI in blue color. Bars = 20 μm.

The isolated SC culture was 95% pure as indicated by immunostaining for AMH (Figure 1a) with an extremely low percentage of non-SC cells (<5%) characterized by immunostaining for insulin-like 3-positive (Leydig) cells (INSL-3) (Figure 1b), alpha-smooth muscle actin positive (peritubular myoid) cells (ASMA) (Figure 1c) and protein gene product 9.5-positive (gonocytes and spermatogonial) cells (PGP9.5) (Figure 1d).

### Inhibin B, AMH, and FSH-r Gene Expression in SC

**Figure 2:**
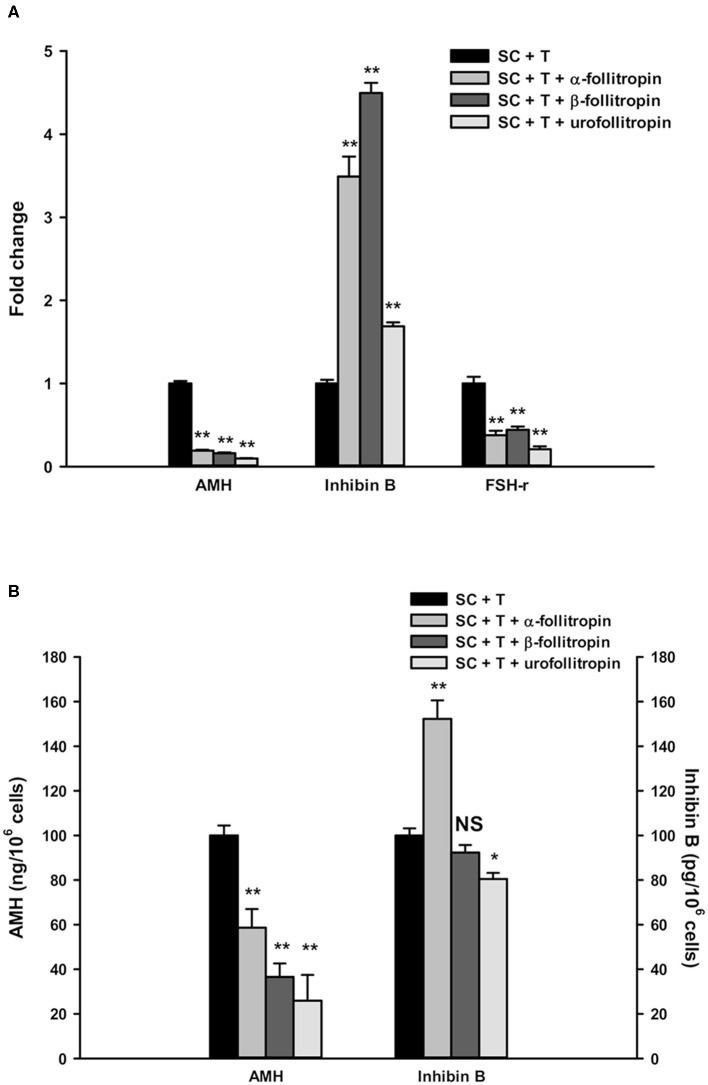
Real-time PCR analysis and ELISA assays. **(A)** Gene expression in SC of AMH, inhibin B, and FSH-r. **(B)** AMH and inhibin B secretion in SC culture medium. Data represent the mean ± S.D. (*p < 0.05, **p < 0.001) of three independent experiments performed in triplicate.

AMH gene expression in SC was significantly down-regulated by treatment with α-, β-follitropin, and urofollitropin in combination with testosterone treatment compared with testosterone alone (Figure 2A, *p* < 0.001).

In contrast, inhibin B expression was significantly increased after treatment with α-, β-follitropin, and urofollitropin, each other combined with testosterone treatment compared with testosterone alone (Figure 2A, *p* < 0.001).

Moreover, we found a statistically significant reduction of FSH-r upon all three FSH preparations plus testosterone compared with testosterone alone (Figure 2A, *p* < 0.001).

### Inhibin B and AMH Secretion Assay

The secretion of AMH was significantly down-regulated by α-, β-follitropin, and urofollitropin plus testosterone treatments compared with testosterone alone, consistent with the results of gene expression showed above (Figure 2B, *p* < 0.001).

Meanwhile, inhibin B was significantly increased in culture medium only after exposure to α-follitropin plus testosterone (Figure 2B, *p* < 0.001), while no changes were observed after β-follitropin plus testosterone stimulation. Interestingly, we observed a significant reduction of inhibin B after the stimulation with urofollitropin plus testosterone compared with testosterone alone (Figure 2B, *p* < 0.05).

### Secretomic Protein Profiling

In order to evaluate the differences induced by the different FSH preparations on SC secretomic profiles, we performed a comparative proteomic analysis of the SC culture media in the groups treated with testosterone associated with α-, β-follitropin, and urofollitropin, and we compared it with the group of the testosterone treatment alone.

The proteomic analysis resulted in the identification of 46 TMT-labeled proteins in all the SC culture media proteomes (Table 2). Of those, 13 proteins were detected in a significantly altered abundance (Table 3). Specifically, 11 proteins were observed as up-regulated (Table 3; cut-off ≥ 1.500) and 2 proteins as down-regulated (Table 3; cut-off ≤ 0.667) by the different FSH preparations. All the different FSH preparations showed a down-regulation of the secreted SPARC protein and an up-regulation of 3 proteins (PLAT, INHBA, and TPI1, Table 3).

**Table 2:** List of the 46 TMT-labeled proteins identified in the Sertoli cell culture.

**Accession**	**Gene name**	**Description**	**Coverage**	**# Proteins**	**# Peptides**	**# Unique Peptides**	**MW (kDa)**
P08835	ALB	Serum albumin	14.00	1	12	12	69.6
Q29549	CLU	Clusterin	40.36	1	14	14	51.7
P01025	C3	Complement C3	16.68	1	21	21	186.7
P04087	INHA	Inhibin alpha chain	31.04	1	8	8	39.2
Q6QAQ1	ACTB	Actin, cytoplasmic 1	22.40	1	6	2	41.7
P20305	GSN	Gelsolin (Fragment)	11.79	1	7	7	84.7
Q9GKQ6	BGN	Biglycan (Fragments)	31.99	1	7	7	30.4
P00761	N/A	Trypsin	9.96	1	2	2	24.4
P18648	APOA1	Apolipoprotein A-I	32.83	1	7	7	30.3
P02543	VIM	Vimentin	8.37	1	4	4	53.6
P04404	CHGA	Chromogranin-A (Fragment)	16.59	1	5	5	49.3
P20112	SPARC	SPARC	24.67	1	6	6	34.2
P68137	ACTA1	Actin, alpha skeletal muscle	15.65	1	5	1	42.0
P0CG68	UBC	Polyubiquitin-C	32.83	2	2	2	60.0
Q29095	PTGDS	Prostaglandin-H2 D-isomerase	16.40	1	3	3	20.6
Q8SQ23	PLAT	Tissue-type plasminogen activator	8.54	1	3	3	63.6
Q1KYT0	ENO3	Beta-enolase	8.06	1	2	2	47.1
P80031	GSTP1	Glutathione S-transferase P	7.73	1	1	1	23.5
P35624	TIMP1	Metalloproteinase inhibitor 1	9.18	1	1	1	23.1
P62802	N/A	Histone H4	11.65	1	1	1	11.4
A5A8V7	HSPA1L	Heat shock 70 kDa protein 1-like	6.08	1	2	2	70.3
P10668	CFL1	Cofilin-1	18.67	2	2	2	18.5
Q9XSD9	DCN	Decorin	5.28	1	2	2	39.9
Q49I35	LGALS1	Galectin-1	5.93	1	1	1	14.7
P52552	PRDX2	Peroxiredoxin-2 (Fragment)	7.09	1	1	1	14.2
O97763	NPC2	NPC intracellular cholesterol transporter 2	16.78	1	2	2	16.3
P79295	AMH	Muellerian-inhibiting factor	7.65	1	3	3	61.5
P62936	PPIA	Peptidyl-prolyl cis-trans isomerase A	5.49	1	1	1	17.9
Q29243	DAG1	Dystroglycan	1.82	1	1	1	95.4
P14287	SPP1	Osteopontin	5.28	1	1	1	33.6
P00690	AMY2	Pancreatic alpha-amylase	2.74	1	1	1	57.0
P43368	CAPN3	Calpain-3	1.83	1	1	1	94.5
P00172	CYB5A	Cytochrome b5	6.72	1	1	1	15.3
P29412	EEF1B	Elongation factor 1-beta	6.70	1	1	1	24.6
P02067	HBB	Hemoglobin subunit beta	6.12	1	1	1	16.2
O02705	HSP90AA1	Heat shock protein HSP 90-alpha	1.91	1	1	1	84.7
P03970	INHBA	Inhibin beta A chain	7.08	1	2	2	47.4
P01315	INS	Insulin	19.44	1	1	1	11.7
P80928	MIF	Macrophage migration inhibitory factor	7.83	1	1	1	12.4
Q2EN75	S100A6	Protein S100-A6	8.89	1	1	1	10.1
P03974	VCP	Transitional endoplasmic reticulum ATPase	2.11	1	1	1	89.2
Q29371	TPI1	Triosephosphate isomerase	6.05	1	1	1	26.7
P42639	TPM1	Tropomyosin alpha-1 chain	4.58	1	1	1	32.7
P09571	TF	Serotransferrin	2.01	1	1	1	76.9
P50390	TTR	Transthyretin	4.67	1	1	1	16.1
P04185	PLAU	Urokinase-type plasminogen activator	3.85	1	1	1	49.1

*N/A, Not applicable*.

**Table 3:** List of the up- and down-regulated proteins in Sertoli cell medium after stimulation with the different FSH preparations plus testosterone, compared with testosterone treatment alone.

**Accession**	**Gene name**	**Description**	**αFSH+T/T**	**βFSH+T/T**	**uFSH+T/T**
P03970	INHBA	Inhibin beta A chain	**1.804**	**1.835**	**3.084**
Q8SQ23	PLAT	Tissue-type plasminogen activator	**1.881**	**2.533**	**1.857**
Q29371	TPI1	Triosephosphate isomerase	**1.786**	**2.132**	**1.562**
Q6QAQ1	ACTB	Actin, cytoplasmic 1	1.377	**1.618**	1.147
P0CG68	UBC	Polyubiquitin-C	1.185	**1.500**	1.357
P52552	PRDX2	Peroxiredoxin-2 (Fragment)	1.459	**1.634**	1.077
P62936	PPIA	Peptidyl-prolyl cis-trans isomerase A	1.358	**1.980**	1.076
P14287	SPP1	Osteopontin	1.016	**2.134**	1.191
P02067	HBB	Hemoglobin subunit beta	1.270	**1.814**	1.336
P80928	MIF	Macrophage migration inhibitory factor	1.412	**1.513**	1.233
Q2EN75	S100A6	Protein S100-A6	1.302	**1.564**	1.100
P20112	SPARC	SPARC	**0.589**	**0.474**	**0.485**
P62802	N/A	Histone H4	1.275	**0.630**	1.049

*N/A, Not applicable*.

*The values in bold indicate the cut-off ≥1.500 for up-regulated proteins, and ≤ 0.667 for down-regulated proteins*.

Interestingly, an up-regulation of 8 additional secreted proteins (ACTB, UBC, PRDX2, PPIA, SPP1, HBB, MIF, and S100A6) and a down-regulation of Histone H4 were specifically observed after the use of β-FSH with T, as reported in Table 3.

## Discussion

In the present work, we have focused on the “*in vitro*” effect of different FSH preparations on pre-pubertal porcine SC culture, evaluating the modulation of specific markers determined through different approaches. All the FSH preparations assessed in the current study, α-, β-follitropin, and urofollitropin, induced a significant and similar response in terms of down-regulation of both AMH gene expression and AMH secretion, up-regulation of inhibin B gene expression and down-regulation of the expression of FSH-r gene expression. AMH is a glycoprotein dimeric hormone, a member of the transforming growth factor β (TGF-β) family, that plays a pivotal function in fetal sex differentiation, being involved in the regression of the Müllerian ducts (22). In the male, SC secretes high amounts of AMH from fetal life until the onset of puberty. AMH is exclusively secreted by SC and, for this reason, it is widely considered an important marker of the testicular function during the pre-pubertal life (22). We observed that testosterone alone and in combination with the three FSH preparations induced a significant down-regulation in AMH mRNA expression and secretion. As expected, we also demonstrated a statistically significant reduction in FSH-r expression independently of the FSH preparation. These data are in accordance with literature reporting how the interaction of the hormone with its receptor leads to the down-regulation of FSH-r mRNA expression by a cAMP-dependent post-transcriptional mechanism (23).

Another specific and important marker of SC functionality is inhibin B, which provides for a negative feedback on FSH secretion. In particular, serum inhibin B concentration is high during early postnatal life, and then gradually is reduced to a detectable plateau-level until its increase at the beginning of puberty (12). The assay of inhibin B is used in clinical practice to evaluate the presence and function of SC during childhood. Additionally, in adult life, the inhibin B levels depend on the presence of germ cells thus reflecting the efficiency of spermatogenesis (24). Our results demonstrated that all three FSH preparations plus testosterone significantly induced an up-regulation in the levels of inhibin B mRNA, confirming the role of FSH in inducing the transcription of inhibin B gene.

Our SC secretomic analysis uncovered a similar response after stimulation of SC with the different FSH preparations. On the one hand, we demonstrated the reduction levels of secreted SPARC protein after stimulation of SC with the different FSH preparations. SPARC, also known as osteonectin or BM-40, is a multifunctional protein that can modulate cell shape, proliferation, differentiation, and migration (25). SPARC has been found to interact with structural matrix proteins and may act to mediate their interactions with cells (24–28). In addition, SPARC can regulate the activity of several signaling molecules, either by direct interaction or by interfering with their signaling pathways (29–31). In our scenario, it is known that SPARC is produced by Leydig and Sertoli cells (32, 33), and that it is internalized in Sertoli, Leydig, and germ cells (34), playing a paracrine regulatory role during fetal testis development. Furthermore, the expression of SPARC in SC bearing late-stage elongate spermatids might suggest a role in the spermiation of elongated spermatids (33). Future studies will comprehensively define the function of SPARC in Sertoli-germ cell interaction and spermiogenesis. Here we provide for the first time information about the down-regulation of SPARC secretion by FSH.

On the other hand, the proteomic analysis also showed that tissue-type plasminogen activator (PLAT), triosephosphate isomerase (TPI1), and inhibin beta A chain (INHBA) proteins were up-regulated by the stimulation with α-, β-follitropin, and urofollitropin in presence of normal androgen milieu. Specifically, the inhibin beta A chain was observed as upregulated in proteomics by all the FSH preparations, and the higher increase was observed for urofollitropin. Despite this evidence, only α-follitropin stimulation induced a significative increase in the inhibin B levels in the medium, as documented by ELISA. INHBA is a subunit of both activin B and inhibin B (24). In the testis, it has been postulated that activin B acts as an autocrine and paracrine regulator of spermatogenesis (35, 36), modulating the proliferation in the testis of germ cells and SC (37). We might speculate that β-follitropin and, especially, urofollitropin, induce the activation of the INHB gene and the production of INHB, but do not increase the levels of INHB in the medium since they might increase the levels of activin B instead of inhibin B. Further studies are so needed to understand how the different FSH preparations modify the inhibin B and activin B balance.

The remaining two up-regulated proteins have been previously associated with SC. For example, previous studies demonstrated low levels of PLAT activity in cultured SC under basal conditions, whereas FSH stimulation induces PLAT (38, 39). Our results through a quantitative *in vitro* secretomic approach support the increased secretion of PLAT after FSH treatment independently of the FSH preparation. Interestingly, the plasminogen activator system acts in the process of spermiation (40), the detachment of residual bodies from the mature spermatids (41), and the residual body phagocytosis by SC (42). Triosephosphate isomerase has moreover been previously reported to be expressed in SC (43). An increased TPI1 expression in SC may influence the early activities of spermatogenesis, such as mitosis or initiation of meiosis, by spermatogonia or pre-leptotene spermatocytes, respectively (43).

Surprisingly, the combination of β-follitropin with testosterone revealed specific effects in the SC function besides the aforementioned similar effect of all tested FSH treatments. Specifically, the levels of eight additional proteins were up-regulated, which were: Actin (ACTB), Polyubiquitin-C (UBC), Peroxiredoxin-2 (PRDX2), Peptidyl-prolyl cis-trans isomerase A (PPIA), Osteopontin (SPP1), Hemoglobin subunit beta (HBB), Macrophage migration inhibitory factor (MIF), and Protein S100-A6 (S100A6); and just one protein, Histone H4, was down-regulated.

It is important to underline that some of these proteins might have a pivotal role both in the germ cell migration and in the cell-to-cell contact at the blood-testis barrier (BTB). For example, actin filament bundles have been described in specific Sertoli cell regions that are adjoining to tight junctions and to the sites of adhesion to spermatogenic cells (44). During spermatogenesis, these actin bundles undergo organizational changes, which might play a role in changing the interrelationship between SC and germ cells by facilitating the movement of spermatogenic cells (44). Similarly, SPP1, synthesized by SC and germ cells, is involved in cell adhesion and migration (45), and MIF, produced by SC under basal conditions, induces the migration of spermatogonial cells (46). Also, we found up-regulation of PPIA, a protein highly expressed in SC that has been recognized as a crucial factor in BTB integrity and maintenance (47), as well as S100A6, a protein that promotes cell migration and influences cell junction of SC (48) that could be implied in the spermatogenesis and modulation of BTB.

Interestingly, β-follitropin also increased PRDX2, an antioxidant protein preferentially expressed in SC that might play a role in removing or regulating the intracellular levels of peroxides produced during metabolism (49, 50). Additional up-regulated proteins were HBB, a metalloprotein that acts as a scavenger balancing the level of carbon monoxide (CO) in testis (51), and UBC, which is required for normal spermatogenesis development (52).

In conclusion, α-, β-follitropin, and urofollitropin induced a similar response as expressed by the down-regulation of AMH gene expression and AMH secretion, and an up-regulation and down-regulation of inhibin B and FSH-r gene expression, respectively, thus exerting an interesting effect in inducing maturation of SC from a pre-pubertal to an adult phenotype. This data confirms that the FSH in presence of an androgenic milieu regulates the proliferation and functional maturation of Sertoli cell type (53). Moreover, all three FSH preparations induced a down-regulation of a spermiation related protein, the SPARC, supporting the role of FSH in the regulation of spermatogenesis. Nevertheless, there are some specific effects of each FSH preparation, which need consideration before their prescription to infertile males.

For instance, only α-follitropin, in association with testosterone, induced the secretion in the media of inhibin B. Since inhibin B secreted by Sertoli cells could serve as negative feedback control on the hypothalamic-pituitary system to decrease FSH release (54), we might suppose that α-follitropin could have an inhibitory effect on the hypothalamic/pituitary axis *in vivo*. If these preliminary data obtained in our *in vitro* model would be confirmed by further *in vitro* and *in vivo* studies, we might conclude that male secondary hypogonadism would represent the best indication for this treatment since it induces a good response in terms of Sertoli activation, and the increase in Inhibin B secretion does not have any clinical relevance. In contrast, the increase of Inhibin B secretion as a response to α-follitropin treatment might represent a problem for normogonadotropic infertile patients since it might inhibit physiological pituitary FSH secretion.

In the case of urofollitropin, it induces a similar secretomic profile but also an increase in the release of INHBA without increasing the levels of secreted inhibin B, suggestive of an increasing release of activin B. This panel of action might be useful for treating oligozoospermic patients with normal FSH secretion. Therefore, we might speculate that—if these preliminary data would be confirmed—urofollitropin might represent the best treatment option for patients with normogonadotropic hypogonadism, since it seems to induce an increase in the secreted proteins, including the A chain of inhibin B and, consequentially, activin B, without increasing the levels of inhibin B, which might interfere with pituitary FSH secretion.

Finally, β-FSH stimulation exhibits additional effect up-regulating specific proteins mainly related to spermatogenic cell migration and BTB maintenance. In this context, the stimulation of SC with β-follitropin exerts his effect in the modulation of additional proteins implicated in the last stage of spermiation and in the related antioxidant activity. Further studies are needed to confirm that β-follitropin treatment is the best treatment option for patients with a spermatogenic arrest at the spermatid stage, as suggested by these preliminary *in vitro* data.

This molecular and proteomic approach demonstrated how some molecular effects seem to have a specific signature depending on each FSH preparation. The different molecular responses could help to choose which of the different FSH preparations could be used for infertility treatment according to the different present physio-pathological conditions. We can argue that α-follitropin can find a specific clinical use in hypospermatogenesis due to hypogonadotropic stimulus or in inducing spermatogenesis in puberty; β-follitropin could be specifically indicated to improve spermiation or in case of spermatidic arrest; and, finally, urofollitropin could be useful in idiopathic infertility in normogonadotropic patients. In conclusion, we performed for the first time a comparative study on the effects of different preparations of FSH on *in vitro* porcine pre-pubertal SC model. In the landscape of a personalized medicine, this study opens a window on the different use of the FSH formulations in relation to various clinical therapeutic targets.

## Data Availability Statement

All datasets presented in this study are included in the article/supplementary material.

## Ethics Statement

The animal study was reviewed and approved by Italian Approved Animal Welfare Assurance (A-3143-01).

## Author Contributions

All authors had critically revised and approved the final version of the manuscript. IA, GG, and FB designed and drafted the manuscript. The experimental procedures and data analysis were performed by GG, FB, CB, CL, MJ, MA, FV, and FManci. AP, RC, and RO gave experimental guidance. GL, FMancu, and DM revised the manuscript.

## Conflict of Interest

The authors declare that the research was conducted in the absence of any commercial or financial relationships that could be construed as a potential conflict of interest. The handling Editor declared a past co-authorship with the authors IA, CL, GL, FM, and RC.
